# Psychotic experiences, emotion regulation, and suicidal ideation among Chilean adolescents in the general population

**DOI:** 10.3389/fpsyt.2022.983250

**Published:** 2022-11-16

**Authors:** Heather M. Wastler, Daniel Núñez

**Affiliations:** ^1^Department of Psychiatry and Behavioral Health, The Ohio State University, Columbus, OH, United States; ^2^Faculty of Psychology, Universidad de Talca, Talca, Chile; ^3^Millennium Nucleus to Improve the Mental Health of Adolescents and Youths (Imhay), Santiago, Chile; ^4^Programa de Investigación Asociativa, Faculty of Psychology, Centro de Investigación en Ciencias Cognitivas, Universidad de Talca, Talca, Chile

**Keywords:** psychotic experiences, psychosis proneness, suicide risk, adolescence, developing countries

## Abstract

Psychotic experiences are associated with increased risk for suicide. Despite this well-established finding, very little is known about factors that contribute to this relationship. The current study investigated the relationship between psychotic experiences, emotion regulation, and suicidal ideation among 1,590 Chilean adolescents in the general population. Participants completed self-report measures of psychotic experiences (Community Assessment of Psychic Experiences), emotion regulation (Emotion Regulation Questionnaire), depression (Patient Health Questionnaire-9), and suicidal ideation (Columbia Suicide Severity Rating Scale). Statistical analyses included Mann–Whitney U tests, point-biserial correlations, logistic regression, and moderation analyses. Results suggest that paranoid ideation, bizarre experiences, and perceptual abnormalities were moderately associated with suicidal ideation. Additionally, greater expressive suppression and cognitive reappraisal were associated with suicidal ideation. Results from the logistic regression indicate that paranoid ideation, perceptual abnormalities, and expressive suppression have the strongest relationship with suicidal ideation, even when controlling for depression and relevant demographic variables. Additionally, paranoid ideation interacted with expressive suppression to predict suicidal ideation, with expressive suppression having the strongest relationship with suicidal ideation when paranoid ideation was low to moderate. Taken together, these findings support the broader literature suggesting that emotion regulation might be a transdiagnostic risk factor for suicidal ideation. Additional longitudinal research is needed to examine whether expressive suppression and other maladaptive emotion regulation strategies serve as a mechanism for suicidal ideation both in the general population and among individuals with psychotic experiences.

## Introduction

Psychotic disorders are serious mental illnesses that affect approximately 7.49/1,000 persons worldwide (1). There is growing recognition that psychosis exist on a continuum, ranging from subthreshold psychotic experiences to chronic mental health conditions such as schizophrenia spectrum disorders. Even among individuals who do not meet diagnostic criteria for a psychotic disorder, the presence of psychotic experiences is associated with significant distress (2, 3) and functional impairment (3). Importantly, approximately 7.5% of adolescents have psychotic experiences (3, 4), with such experiences increasing the risk of developing psychotic disorders and other mental health conditions later in life (2, 5–8). Additionally, psychotic experiences are associated with a number of co-occurring mental health difficulties, such as substance use (9, 10), depression (11, 12), anxiety (11, 12), greater emotional reactivity (13, 14), and trauma (15). There is also a growing body of literature demonstrating that psychotic experiences are associated with increased risk for suicide (16, 17). Specifically, recent meta-analyses have shown that psychotic experiences confer increased risk for suicidal ideation, suicide attempts, and suicide (16, 17). Interestingly, the strongest support has been for an association between positive symptoms (i.e., delusions and hallucinations) and suicide risk (16), with studies showing that specific psychotic experiences, such as persecutory ideation and perceptual abnormalities (PA) (18) might have the strongest association with suicide risk. Despite this growing body of literature, much less is known about factors that contribute to the relationship between psychotic experiences and suicide risk.

Outside of the psychosis literature, emotion regulation, the use of strategies to change the intensity, frequency, or perceived quality of emotion (19, 20), has been identified as a trans-diagnostic mechanism for suicide risk (21–24). Emotion regulation abnormalities are well documented among individuals with psychosis, with studies showing that individuals with schizophrenia spectrum disorders use greater maladaptive emotion regulation strategies (e.g., rumination, self-blaming, distraction, and suppression) and fewer adaptive strategies (e.g., reappraisal) than healthy controls (25, 26). Importantly, emotion regulation abnormalities exist across the psychosis continuum (27), occurring among adolescents with psychotic experiences (28, 29), individuals at clinical high-risk for psychosis (14, 30), and among individuals diagnosed with schizophrenia spectrum disorders (25, 26). Although few studies have explicitly examined how emotion regulation abnormalities can affect psychotic experiences, studies have shown that maladaptive emotion regulation strategies might maintain and exacerbate psychotic experiences (31). For instance, a recent study demonstrated that adaptive strategies such as cognitive reappraisal are used similarly among individuals with high/low psychotic experiences, whereas maladaptive strategies, such as expressive suppression, are more common among individuals with greater psychotic experiences (32). Further, another study demonstrated that individuals with psychotic disorders experience more difficulty regulating their emotions during the presence of psychotic experiences (33). Taken together, these findings suggest an important relationship between psychotic experiences and emotion regulation, highlighting the need for more research in this area (33).

Despite the well-established relationship between emotion regulation abnormalities and psychotic disorders, limited research has examined whether emotion regulation abnormalities contribute to suicidal risk in this population. This is a notable gap in the literature, as emotion regulation plays in important role across leading suicide theories (e.g., Escape Theory, Interpersonal Psychological Theory, Three Step Theory, Integrated Motivational-Volitional Model, and Fluid Vulnerability Theory) (34–38) and several theories even explicitly identify emotion regulation as a mechanism for suicidal thoughts and behaviors (23, 24, 39). The few studies that have examined emotion regulation and suicide risk in psychosis have found that emotion dysregulation (40) and coping beliefs (i.e., the perceived ability to regulation emotions) (41, 42) are associated with suicide risk among individuals with psychosis. Only one study to date has explicitly examined whether the use of maladaptive versus adaptive emotion regulation strategies contributes to the relationship between psychotic experiences and suicide risk (43). This study found that auditory hallucinations are associated with suicidal behavior and that lower reappraisal mediates this relationship; expressive suppression did not mediate the relationship between psychotic experiences and suicidal behavior. Taken together, these studies provide preliminary evidence that emotion regulation might influence the relationship between suicide risk and psychotic experiences. Notably, however, these studies were primarily based on high-income Western countries (e.g., Australia, United States, and England), which limits our understanding about factors that contribute to suicide among adolescents with psychotic experiences from developing countries (40, 41, 43). This is a significant gap in the literature as (1) psychotic experiences are more common in lower and middle income countries than high income countries (44); (2) suicide is a leading cause of death among adolescents worldwide (45); and (3) the consequences of specific emotion regulation strategies is culturally dependent (46). Thus, there is a great need for further research examining the relationship between psychotic experiences, emotion regulation, and suicidal ideation among adolescents from developing countries.

The current study sought to address this gap by examining the cross-sectional relationship between psychotic experiences, emotion regulation, and suicidal ideation among Chilean adolescents in the general population. The current study focuses specifically on adolescence, as suicide is a leading cause of death among adolescents worldwide (47). Additionally, we focus on Chilean adolescents, as prior research has shown that 52.5% of Chilean adolescents endorse bizarre experiences (BE) and 15.3% endorse perceptual anomalies in their lifetime, suggesting that psychotic experiences are common and relevant for this population (48). Based on prior studies, we hypothesized that greater psychotic experiences, specifically PA and paranoid ideation (PI) (18), would be associated with suicidal ideation. We also hypothesized that greater use of maladaptive emotion regulation strategies (i.e., expressive suppression) and decreased use of adaptive emotion regulation strategies (i.e., cognitive reappraisal) would be associated with suicidal ideation. Finally, we hypothesized that psychotic experiences would interact with emotion regulation to predict suicidal ideation, such that individuals with greater psychotic experiences and greater expressive suppression would have the highest rates of suicidal ideation.

## Methods

### Participants and procedures

Participants included 1,590 adolescents recruited from secondary public schools in Chile from April to September 2019. Participants were recruited from parents’ meetings, where they received information about the project before making a decision about their adolescent’s participation. Inclusion criteria were as follows: (1) ages 12–19 years and (2) written consent provided by both the adolescent and parent/legal guardian. Participants were only excluded if they were outside of the ages of 12–19 years. Participants then completed the self-report questionnaires administered by trained psychologists in a classroom setting. This study was approved by the Bioethics Committee of the University of Talca (02-2021).

### Measures

#### Psychotic experiences

The Community Assessment of Psychic Experiences (CAPE-P15) (49) was used to assess psychotic experiences. The CAPE-P15 is a 15 items self-report measure that assesses BE (7 items), PA (3 items), and PI (5 items). Items were rated on a *1 (never)* to *5 (very often)* Likert scale (total scores ranging from 15 to 75), with higher scores indicating greater frequency of psychotic experiences. Cronbach’s alpha for the entire sample was 0.80 for PA, 0.82 for PI, and 0.85 for BE.

#### Suicidal ideation

The Columbia Suicide Severity Rating Scale (C-SSRS) (50) was used to assess suicidal ideation. Consistent with prior studies, items were adapted and administered in a self-report format (51). This self-report version included 7-items that assess suicidal ideation, suicide planning, and preparatory/suicidal behavior (51). Prior research has demonstrated that suicidal ideation and resolved planning/preparation are two distinct constructs (52–56) and that there are distinct risk factors for suicidal ideation and behavior (57, 58). Due to the higher prevalence of suicidal ideation, the current study focused only on suicidal ideation. Two items were used to assess suicidal ideation: (1) *Have you wished you were dead or wished you could go to sleep and not wake up* and (2) *Have you actually had any thoughts of killing yourself?* These items were used to create a binary variable for the presence of suicidal ideation.

#### Emotion regulation

The Emotion Regulation Questionnaire (ERQ) (59) was used to assess emotion regulation. The ERQ is a 10 item self-report measure that assesses the habitual use of cognitive reappraisal (i.e., reinterpreting an emotional event to change an emotion) and expressive suppression (i.e., concealing the outward expression of emotions). Items were rated on a *1 (strongly disagree)* to *7 (strongly agree)* Likert scale, with higher scores indicating greater use of each strategy. Cronbach’s alpha for the entire sample was 0.83 for cognitive reappraisal and 0.71 for expressive suppression.

#### Depressive symptoms

The Patient Health Questionnaire-9 (PHQ-9) (60) was used to assess depressive symptoms. The PHQ-9 is a 9-item self-report questionnaire with items rated on a *0 (not at all)* to *3 (nearly every day)* scale. Total scores range from 0 to 27 with scores 0–4 indicating no depressive symptoms, scores 5–9 indicating mild depressive symptoms, scores 10–14 indicating moderate depressive symptoms, scores 15–29 moderately severe depressive symptoms, and 20–27 indicating severe depressive symptoms. One PHQ-9 item assesses thoughts about wanting to be dead/thoughts of hurting oneself. To prevent criterion contamination, we calculated a total depression score excluding the suicidal ideation item of the PHQ-9. Cronbach’s alpha for the entire sample was 0.89.

### Statistical analyses

First, we used descriptive statistics to examine the frequency of lifetime suicidal ideation in our sample. Variables of interest were not normally distributed. Therefore, Mann–Whitney U tests were used to examine whether individuals with and without suicidal ideation differed in psychotic experiences, emotion regulation, and depression. We then used point-biserial correlations to examine the relationship between suicidal ideation, depression, psychotic experiences, and emotion regulation. Multivariate logistic regression was used to determine which variables had the strongest relationship with suicidal ideation. Prior to conducting the logistic regression, we used Chi-square and point-biserial correlations to determine whether age and gender would be included in our model. Using *a priori* criteria (*p* < 0.10), we determined that both age and gender would be included as covariates. Logistic regression assumptions were also examined; 22 participants were identified as multivariate outliers and were therefore excluded from the analysis. We also conducted follow-up moderation analyses to examine the relationship between emotion regulation and suicidal ideation among individuals with psychotic experiences. Two separate logistic regression models were conducted with an interaction term for PI/expressive suppression in model 1 and PA/expressive suppression in model 2; multivariate outliers were excluded from each moderation analysis. Simple effects were used to probe significant interaction terms (61, 62).

## Results

### Participant characteristics and descriptive statistics

Our sample included 1,590 (751 females, 835 males, 4 with no sex reported) Chilean adolescents. The mean age was 15.26 ± 1.35. Lifetime suicidal ideation was present in 48.2% (*n* = 767) of the sample. Preparatory/suicidal behavior was present in only 2.8% (*n* = 45) of the total sample. Depressive symptoms (*p* < 0.001), PI (*p* < 0.001), BE (*p* < 0.001), and PA (*p* < 0.001) were greater among adolescents with suicidal ideation (Table 1). Additionally, adolescents with lifetime suicidal ideation endorsed greater habitual use of expressive suppression (*p* = 0.001) and cognitive reappraisal (*p* < 0.001) (Table 1).

**TABLE 1:** Depression, psychotic experiences, and emotion regulation among adolescents with and without suicidal ideation.

	No SI (*n* = 823)	SI (*n* = 767)			
	*M* (SD)	*M* (SD)	*U*	*p*	η ^2^
Depressive symptoms	5.04 (3.82)	10.88 (5.51)	514,921.50	<0.001	0.300
Paranoid ideation	8.25 (2.85)	11.38 (4.14)	468,729.00	<0.001	0.178
Bizarre experiences	9.31 (3.06)	12.74 (5.41)	453,204.00	<0.001	0.145
Perceptual abnormalities	3.66 (1.41)	4.67 (2.38)	404,323.00	<0.001	0.075
Expressive suppression	15.00 (5.76)	18.04 (4.90)	413,180.00	0.001	0.072
Cognitive reappraisal	26.51 (8.74)	28.35 (7.18)	345,666.00	<0.001	0.007

### Correlation analysis

Point-biserial correlations between suicidal ideation, depression, psychotic experiences, and emotion regulation are displayed in Table 2. Suicidal ideation had a moderate association with depression (*p* < 0.001), PI (*p* < 0.001), BE (*p* < 0.001), PA (*p* < 0.001), and expressive suppression (*p* < 0.001). The relationship between suicidal ideation and cognitive reappraisal was significant, but small in magnitude.

**TABLE 2:** Relationship between suicidal ideation, depression, psychotic experiences, and emotion regulation.

	SI	PHQ9	CAPE-PI	CAPE-BE	CAPE-PA	ERQ-Cog
PHQ9	0.526**	–				
CAPE-PI	0.405**	0.644**	–			
CAPE-BE	0.366**	0.613**	0.608**	–		
CAPE-PA	0.251**	0.381**	0.396**	0.477**	–	
ERQ-Cog	0.113**	0.077**	0.123**	0.089**	0.012	–
ERQ-Supp	0.272**	0.310**	0.259**	0.268**	0.170**	0.346**

**Correlation significant at the 0.01 level (two-tailed). SI, suicidal ideation; PHQ9, depression; CAPE-PI, paranoid ideation; CAPE-BE, bizarre experiences; CAPE-PA, perceptual abnormalities; ERQ-Cog, cognitive reappraisal; ERQ-Supp, expressive suppression. Correlations between SI and variables of interest were conducted using point-biserial correlations. All other values represent Spearman correlations.

### Logistic regression

Table 3 summarizes results from the logistic regression. The full model including all predictors was significant χ^2^(8) = 691.58, *p* < 0.001], correctly identifying 75.7% of cases and accounting for 35.7% (Cox and Snell *R*^2^) to 47.6% (Nagelkerke *R*^2^) of variance in lifetime suicidal ideation. Regarding demographic variables, older participants had greater odds of experiencing lifetime suicidal ideation and males had a lower odds of experiencing suicidal ideation relative to females. Depression (OR = 1.277, 95% CI = 1.226–1.330), PI (OR = 1.072, 95% CI = 1.019–1.128), and PA (OR = 1.118, 95% CI = 1.025–1.220) were associated with the presence of suicidal ideation. Additionally, greater use of expressive suppression (OR = 1.063, 95% CI = 1.035–1.092), but not cognitive reappraisal, was associated with suicidal ideation.

**TABLE 3:** Logistic regression examining the relationship between psychotic experiences, depression, emotion regulation, and suicidal ideation (*n* = 1,568).

					95% CI	
	*B*	SE	*p*	OR	LL	UL
Age	0.098	0.049	0.044	1.103	1.003	1.214
Sex (female reference group)	−0.498	0.131	<0.001	0.608	0.470	0.786
Depression	0.244	0.021	<0.001	1.277	1.226	1.330
Paranoid ideation	0.070	0.026	0.007	1.072	1.019	1.128
Bizarre experiences	0.035	0.024	0.140	1.036	0.988	1.086
Perceptual abnormalities	0.112	0.044	0.012	1.118	1.025	1.220
Expressive suppression	0.061	0.014	<0.001	1.063	1.035	1.092
Cognitive reappraisal	0.008	0.009	0.423	1.008	0.989	1.027

### Moderation analyses

**FIGURE 1:**
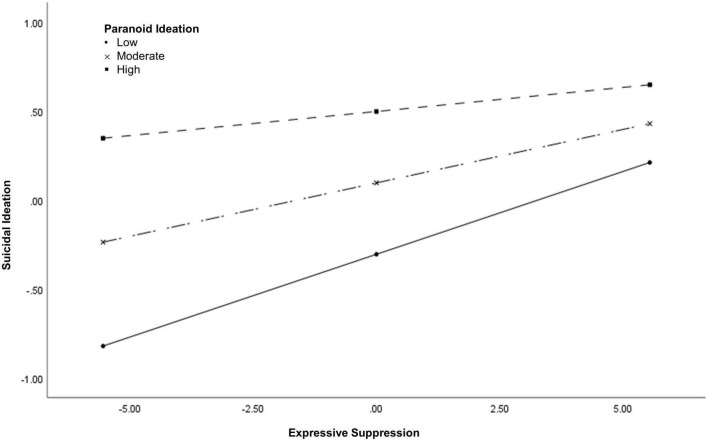
The interaction between paranoid ideation and expressive suppression.

Two separate moderation analyses were used to examine whether emotion regulation interacts with psychotic experiences to predict suicidal ideation. The first model examined the interaction between PI and expressive suppression when controlling for depression. Multivariate outliers were excluded from this analysis (*n* = 1,572). The overall model was significant χ^2^(4) = 670.75, *p* < 0.001], accounting for 34.7% (Cox and Snell *R*^2^) to 46.3% (Nagelkerke *R*^2^) of variance in lifetime suicidal ideation. Paranoid ideation significantly interacted with expressive suppression to predict suicidal ideation even when accounting for depressive symptoms (*B* = −0.009, SE = 0.004, *p* = 0.035, OR = 0.99, 95% CI = 0.98–0.99). Individuals with high levels of PI had the highest rates of expressive suppression and suicidal ideation (Figure 1). However, the relationship between expressive suppression and suicidal ideation was strongest when PI was low (*B* = 0.093, SE = 0.018, *Z* = 5.13, *p* < 0.001, 95% CI = 0.058–0.129) to moderate (*B* = 0.060, SE = 0.013, *Z* = 4.71, *p* < 0.001, 95% CI = 0.035–0.085). There was no relationship between expressive suppression and suicidal ideation when PI was high (*B* = 0.027, SE = 0.022, *Z* = 1.23, *p* = 0.22, 95% CI = −0.016 to 0.070).

The second model examined the interaction between PA and expressive suppression when controlling for depression. Multivariate outliers were excluded from this analysis (*n* = 1,575). The overall model was significant χ^2^(4) = 652.98, *p* < 0.001], accounting for 33.9% (Cox and Snell *R*^2^) to 45.3% (Nagelkerke *R*^2^) of variance in lifetime suicidal ideation. Perceptual abnormalities did not interact with expressive suppression to predict suicidal ideation (*B* = −0.005, SE = 0.008, *p* = 0.506, OR = 0.995, 95% CI = 0.98−1.01). Rather, PA (*B* = 0.149, SE = 0.041, *p* < 0.001, OR = 1.161, 95% CI = 1.072–1.258) and expressive suppression (*B* = 0.060, SE = 0.012, *p* < 0.001, OR = 1.061, 95% CI = 1.036–1.087) were independently associated with suicidal ideation.

## Discussion

The present study examined the relationship between psychotic experiences, emotion regulation, and suicidal ideation among adolescents in Chile. Nearly half of our sample endorsed lifetime suicidal ideation, suggesting that suicide risk is a major concern for this population. Though these rates of suicidal ideation are high, they are consistent with the broader literature demonstrating that approximately 66% of individuals at clinical high-risk for psychosis experience suicidal ideation (63). The current study also found that adolescents with suicidal ideation had higher rates of psychotic experiences and the presence of suicidal ideation was significantly associated with PI, BE, and PA. These findings are consistent with the broader literature (16, 17), suggesting that psychotic experiences, even among individuals who do not meet diagnostic criteria for a schizophrenia spectrum disorder, are associated with increased risk for suicide. Further research is needed to determine the mechanisms by which psychotic experiences confer increased risk for suicide.

Emotion regulation has repeatedly been identified as a trans-diagnostic mechanism for suicidal thoughts and behaviors (21–24). The current study found that adolescents with suicidal ideation report greater habitual use of both adaptive and maladaptive emotion regulation strategies compared to adolescents without suicidal ideation. Similarly, suicidal ideation was associated with greater use of both cognitive reappraisal and expressive suppression in the entire sample, though the association between cognitive reappraisal and suicidal ideation was small in magnitude. Our finding that adolescents with suicidal ideation have *increased* use of cognitive reappraisal is somewhat surprising given prior research demonstrating that *decreased* use of adaptive emotion regulation strategies is associated with suicidal ideation among adults (64). Our results indicate that adolescents with suicidal ideation engage in greater overall efforts to regulate their emotions, rather than only using maladaptive emotion regulation strategies. These inconsistent findings might be attributable to differences in emotion regulation among adolescents versus adults (65, 66) and/or across cultures (46). Additionally, it is possible that adolescents with suicidal ideation engage in more emotion regulation because they experience higher levels of negative affect (67) and/or because their emotion regulation efforts are unsuccessful, thereby warranting increased use of various strategies. Furthermore, the relationship between reappraisal and suicidal ideation might differ depending on the severity of these thoughts; for instance, adolescents with lower severity suicidal ideation might use reappraisal more often than those with higher severity suicidal ideation. Despite these somewhat surprising findings, results from the overall logistic regression model suggest that cognitive reappraisal is no longer associated with suicidal ideation when including psychotic experiences, expressive suppression, depression, and demographic variables in the model. In other words, expressive suppression has a stronger relationship with suicidal ideation than cognitive reappraisal. Although these findings provide preliminary support for the notion that some emotion regulation strategies have a stronger relationship with suicide risk than others, further research is needed to examine other emotion regulation strategies, such as distraction, rumination, emotion suppression, seeking social support, physiological intervention, acceptance (19, 20).

Given the well-established finding that emotion regulation abnormalities exist across the psychosis continuum (14, 25–30), the current study also examined whether psychotic experiences interact with emotion regulation to predict suicidal ideation. Results demonstrate that PI, but not PA, interact with expressive suppression to predict suicidal ideation. Consistent with our hypotheses, individuals with high levels of PI had the highest rates of expressive suppression and suicidal ideation. However, the relationship between expressive suppression and suicidal ideation was actually strongest when PI was low to moderate; additionally, there was no relationship between expressive suppression and suicidal ideation when PI was high. Importantly, expressive suppression involves concealing the outward expression of negative emotions. In the general population, expressive suppression is associated with a wealth of negative consequences, including high blood pressure (68), poor social relationships (68), and suicidal ideation (64). However, for individuals with PI, concealing the outward expression of negative emotions toward others might actually be adaptive, protecting against the negative impact paranoia has on social relationships (69–71). Further, the negative consequences of expressive suppression might be dependent on the specific emotion that an individual is concealing. For instance, concealing the outward expression of paranoia might be adaptive for individuals with psychotic experiences, whereas concealing other emotions such as sadness, anxiety, and anger might lead to negative consequences similar to what is observed in the general population (68). This notion is consistent with broader emotion regulation theories, suggesting that the distinction between adaptive and maladaptive strategies is context dependent and that flexible implementation of various strategies might be key for successful emotion regulation (19, 72). Additional research is needed to determine whether specific emotion regulation strategies are adaptive versus maladaptive within the context of psychotic experiences.

Emotion regulation is widely regarded as a transdiagnostic process, with emotion regulation difficulties presenting across a range of mental health disorders (73, 74). Our findings further support emotion regulation as a transdiagnostic risk factor for suicide as we found that (1) PA and expressive suppression were independently associated with suicidal ideation and (2) that the relationship between expressive suppression and suicidal ideation was strongest when PI was low to moderate. In other words, expressive suppression was not a psychosis specific risk factor for suicidal ideation. Nonetheless, we found that individuals with psychotic experiences engaged in high levels of expressive suppression, which might contribute to the high rates of suicidal ideation in this population. Future longitudinal research is needed to determine whether emotion regulation is a mechanism for suicide risk both in the general population and among individuals with psychotic experiences.

Notable strengths of the current study include (1) our large representative sample, (2) our focus on psychotic experiences among Chilean adolescents, expanding our understanding about suicidal ideation among adolescents from developing countries, and (3) our dimensional approach to assessing psychotic experiences. The current study was limited in the following ways. First, this study involved a cross-sectional design, which precludes our ability to make inferences about the causal nature and direction of the relationship between emotion regulation, psychotic experiences, and suicidal ideation. For instance, it is possible that the relationship between emotion regulation and suicidal ideation is bidirectional, as difficulty regulating negative emotions might lead to suicidal ideation and individuals might engage in emotion regulation strategies to manage suicidal ideation. Research that utilizes ecological momentary assessment would shed light into the temporal patterns associated with emotion regulation and suicidal ideation among individuals with psychosis. Second, the current study focused only on suicidal ideation as an outcome, limiting our understanding about the relationship between psychotic experiences, emotion regulation, and suicidal behavior. Leading suicide theories propose distinct mechanisms for suicidal ideation and suicidal behavior (23, 35, 75). Thus, additional research is needed to examine the relationship between psychotic experiences, emotion regulation, and suicidal behavior. Third, although we recruited from the general population, our sample was limited to adolescents with a relatively narrow age range, limiting the generalizability of our findings. As the onset of psychotic disorders often occurs during late adolescents through young adulthood, additional research that includes young adults is warranted. Finally, the current study did not examine distress associated with psychotic experiences. There is some debate about whether the association between psychotic experiences and suicidal ideation is attributable to third variables such as psychological distress (17). Additional research is needed to determine whether distress influences the relationship between psychotic experiences, emotion regulation, and suicidal ideation.

## Conclusion

In summary, the current study found that psychotic experiences and emotion regulation were associated with suicidal ideation among Chilean adolescents. We found that PI, PA, and expressive suppression had the strongest relationship with suicidal ideation, even when accounting for depression and demographic variables. Although PI and expressive suppression interacted to predict suicidal ideation, results from the simple effects suggested that expressive suppression had a stronger relationship with suicidal ideation among individuals with low to moderate PI. Taken together, these findings suggest that expressive suppression is likely a transdiagnostic risk factor for suicidal ideation and that individuals with psychotic experiences might experience higher rates of suicidal ideation because they engage in greater expressive suppression. Additionally, longitudinal research is needed to further examine whether expressive suppression and other maladaptive emotion regulation strategies (i.e., distraction, emotion suppression, and rumination) serve as a mechanism for suicidal ideation both in the general population and among individuals with psychotic experiences.

## Data availability statement

The raw data supporting the conclusions of this article will be made available by the authors, without undue reservation.

## Ethics statement

The studies involving human participants were reviewed and approved by the Bioethics Committee of the University of Talca. Written informed consent to participate in this study was provided by the participants or their legal guardian/next of kin.

## Author contributions

DN and HW designed the study. HW conducted the literature search, statistical analyses, and prepared the manuscript. DN provided consultation for statistical analyses and critically revised the manuscript for intellectual content. Both authors contributed to the article and approved the submitted version.
